# Implant stability following septal expansion in immediate mandibular molars: a pilot randomized clinical trial comparing piezoelectric surgery and osseodensification

**DOI:** 10.1186/s12903-026-09257-8

**Published:** 2026-07-17

**Authors:** Hussien El. H. Hassan, Abdelaziz F. Khlalil, Dina M. N. Metawie

**Affiliations:** Department of Oral and Maxillofacial Surgery, Faculty of Dentistry, Alexandria University, Alexandria, Egypt

## Abstract

**Background:**

Immediate implant placement (IIP) in mandibular molars is challenged by wide extraction sockets and limited septal bone support. This pilot feasibility randomized clinical trial evaluated primary and secondary implant stability following septal expansion using piezoelectric surgery (PISP) compared with osseodensification (OD) using Densah^®^ burs.

**Methods:**

Twenty patients were randomly allocated (1:1) to either PISP group or OD group for immediate mandibular molar implant placement. The primary outcome was implant stability quotient (ISQ), measured immediately and at 3 months. Secondary outcomes included insertion torque, osteotomy preparation time, and postoperative pain assessed using a visual analogue scale (VAS) during the first postoperative week. Normality was evaluated using Shapiro–Wilk tests and Q–Q plots. Between-group comparisons were performed using independent t-tests, while VAS scores were analyzed using Mann–Whitney U and Friedman tests (α = 0.05).

**Results:**

No statistically significant differences were observed between groups in primary or secondary ISQ (*p* > 0.05). Both groups demonstrated a significant increase in ISQ at 3 months compared with baseline (*p* < 0.001). The mean insertion torque was significantly higher in the PISP group (40.33 ± 3.24 Ncm; *p* = 0.004) compared to the OD group (35.00 ± 3.53 Ncm). Osteotomy preparation time was significantly longer in the PISP group (9.00 ± 0.94 min) than in the OD group (6.00 ± 0.82 min) (*p* < 0.001). Postoperative VAS pain scores were significantly lower in the PISP group throughout the first week (*p* < 0.05). All implants achieved clinical stability with no intra- or postoperative complications.

**Conclusions:**

The preliminary findings of this pilot feasibility trial demonstrated that PISP achieves comparable biomechanical stability to OD, establishing its clinical feasibility for immediate molar implant placement in anatomically complex posterior regions.

**Trial registration:**

NCT06957860 ; registered May 5, 2025.

**Supplementary Information:**

The online version contains supplementary material available at 10.1186/s12903-026-09257-8.

## Introduction

Immediate implant placement (IIP) has emerged as an optimal modality for rehabilitating non-restorable teeth owing to its clinical advantages. This approach preserves alveolar bone by attenuating post-extraction resorption, a process driven by physiological osseous remodeling and volumetric ridge shrinkage. Moreover, it enhances prosthetic outcomes through optimized emergence profiles and hygienic contours, thereby reducing plaque accumulation and peri-implant soft tissue complications. The protocol further shortens overall treatment time by eliminating extended healing intervals and improves patient satisfaction via streamlined procedural timelines [1, 2].

However, IIP in freshly extracted molar sockets poses significant clinical challenges, particularly in attaining sufficient primary stability and insertion torque necessary for predictable osseointegration and immediate provisionalization. These challenges stem from anatomical characteristics of post-extraction sites wide socket dimensions, diminished residual bone density, and proximity to vital neurovascular structures such as the inferior alveolar nerve [3–5].

To overcome these limitations, engaging the interradicular septum a site typically characterized by denser and more intact bone compared to the surrounding socket walls has been established as a strategic method for enhancing implant stabilization. Placement within the septum improves initial stability, increases bone-implant contact (BIC), and promotes osseointegration, while also facilitating precise prosthetic restoration by reducing cantilever forces [6, 7]. Nevertheless, a majority of septal regions exhibit insufficient width, conforming to the Type C socket classification as described by Smith and Tarnow [8], thereby necessitating expansion techniques to achieve the more favorable Type A or B .

Osseodensification (OD) represents a transformative approach to implant site preparation, shifting the paradigm from bone excavation to bone compaction. Pioneered by Dr. Salah Huwais, this technique utilizes specially designed Densah^®^ burs (Versah LLC, USA) featuring reverse flutes and a non-cutting tip. When operated in a counter-clockwise rotation, these burs compress and laterally displace bone trabeculae, creating a densified osteotomy wall through trabecular condensation and autografting. This process generates a biomechanically favorable environment characterized by increased bone density and a “spring-back” effect, enhancing primary stability through improved bone-implant contact [9–11]. 

The established biomechanical advantages and documented clinical success of osseodensification in narrow ridge expansion establish this technique as particularly indicated for septal bone expansion in immediate molar implant placement with deficient septum bone width [12].

Piezoelectric surgery pioneered by Prof. Tomaso Vercellotti around the year 2000 [13], utilizes high-frequency ultrasonic vibrations (24–29 kHz) to perform precise and selective osteotomies in implant dentistry. Unlike conventional rotary instruments, its oscillating tip cuts mineralized tissue through microvibrations and hydrodynamic cavitation while preserving adjacent soft tissues and neurovascular structures. This selective action enables enhanced surgical control and safety. Continuous irrigation during the procedure improves visibility and minimizes thermal trauma, this technique also appears to favor early healing responses; several studies suggest that ultrasonic micro-vibrations stimulate osteoblastic activity and angiogenesis, potentially accelerating secondary stability and early bone remodeling compared with rotary instrumentation [14–18].

The Intralift Kit (Acteon, France) is a specialized system designed to apply these principles in clinical practice. It features angled, diamond-coated tips with progressively increasing diameters (1.35–2.80 mm), engineered for procedures including internal sinus elevation, ridge expansion, and precise osteotomy preparation [19, 20]. 

Therefore, this pilot feasibility randomized clinical trial was conducted to compare implant stability following immediate mandibular molar placement using piezoelectric implant site preparation (PISP) versus osseodensification (OD) after septal expansion.

The null hypothesis was that there would be no statistically significant difference between PISP (study group) and OD (control group) regarding implant stability, osteotomy preparation time, and postoperative pain following septal expansion in immediate mandibular molar sites.

### Aim of study

#### The primary aim of this pilot feasibility randomized clinical trial

- To compare primary implant stability following septal expansion using ISQ values immediate post operative.

#### The secondary aims

- To compare the insertion torque values.
- To compare secondary implant stability using ISQ values.
- To compare percentage of expansion between primary and secondary stability.
- To compare Osteotomy preparation time.
- To compare Postoperative pain, recorded using VAS during the first postoperative week.

#### Predefined feasibility criteria

1. *Recruitment feasibility*: Successful enrollment of the target sample (10 patients per group, 20 total) within the planned recruitment window.
2. *Protocol adherence*: Successful completion of the full surgical and follow-up protocol for all enrolled participants.
3. *Retention*: Absence of dropouts or losses to follow-up over the three month observation period.
4. *Safety*: Absence of serious adverse events, including early implant failure, infection, neurosensory disturbance, or wound dehiscence.
5. *Procedural feasibility*: Successful completion of the septal expansion procedure and immediate implant placement in all included sites.
6. *Outcome feasibility*: Achievement of clinically acceptable primary and secondary stability values consistent with successful osseointegration.

## Methods

### Ethical approval and informed consent

This study was conducted in accordance with the ethical principles outlined in the Declaration of Helsinki. Ethical approval was obtained from the Research Ethics Committee of Alexandria University (Approval No. 0716-06/2023; IORG008839). The manuscript has been prepared in accordance with the CONSORT guidelines. The trial was retrospectively registered at ClinicalTrials.gov (NCT06957860; registered May 5, 2025), because patient enrollment began November 2023 [21]. Written informed consent was obtained from all participants after a comprehensive explanation of the study objectives, procedures, potential risks, and benefits.

### Study design

This single-center, parallel-group pilot feasibility randomized clinical trial was performed at the Outpatient Clinic of the Oral and Maxillofacial Surgery Department, Faculty of Dentistry, Alexandria University, Egypt, between November 2023 and August 2024. Twenty patients requiring immediate implant placement (IIP) in mandibular molars were enrolled. Participants were randomized (1:1) assigned to either the piezoelectric implant site preparation (PISP) study group (*n* = 10) or the osseodensification (OD) control group (*n* = 10) using a computer-generated sequence with block allocation (Random Allocation software 1.0). Allocation concealment was assured by sequentially numbered, sealed opaque envelopes opened at the time of surgery. The surgeon could not be blinded, However, all outcome assessors remained blinded to group assignment throughout the trial, including both the Osstell^®^ operator recording ISQ values and the clinician measuring insertion torque values.

### Participants

Twenty patients (aged 18–40 years) requiring extraction of non-restorable mandibular molars and immediate implant placement were enrolled in this study when the following.

inclusion criteria were satisfied.

### Inclusion criteria

1. Presence of septum width ≥ 2.5 mm and residual bone height ≥ 5 mm apical to the socket with intact buccal and lingual bone walls, confirmed via preoperative CBCT [2].
2. Absence of clinical signs of acute periapical infection (e.g., purulent exudate, swelling, or fistulous tract) at extraction [6].
3. ASA I or II classification (healthy or with well-controlled systemic disease).

### Exclusion criteria

1. Uncontrolled systemic diseases compromising healing (e.g., uncontrolled diabetes, bisphosphonate therapy).
2. Smoking (> 10 cigarettes/day).
3. History of head and neck radiotherapy within the preceding 12 months [2, 6].

### Materials

*Implants*: Vitronex^®^ implants (Milano, Italy) [22]

**Fig. 1:**
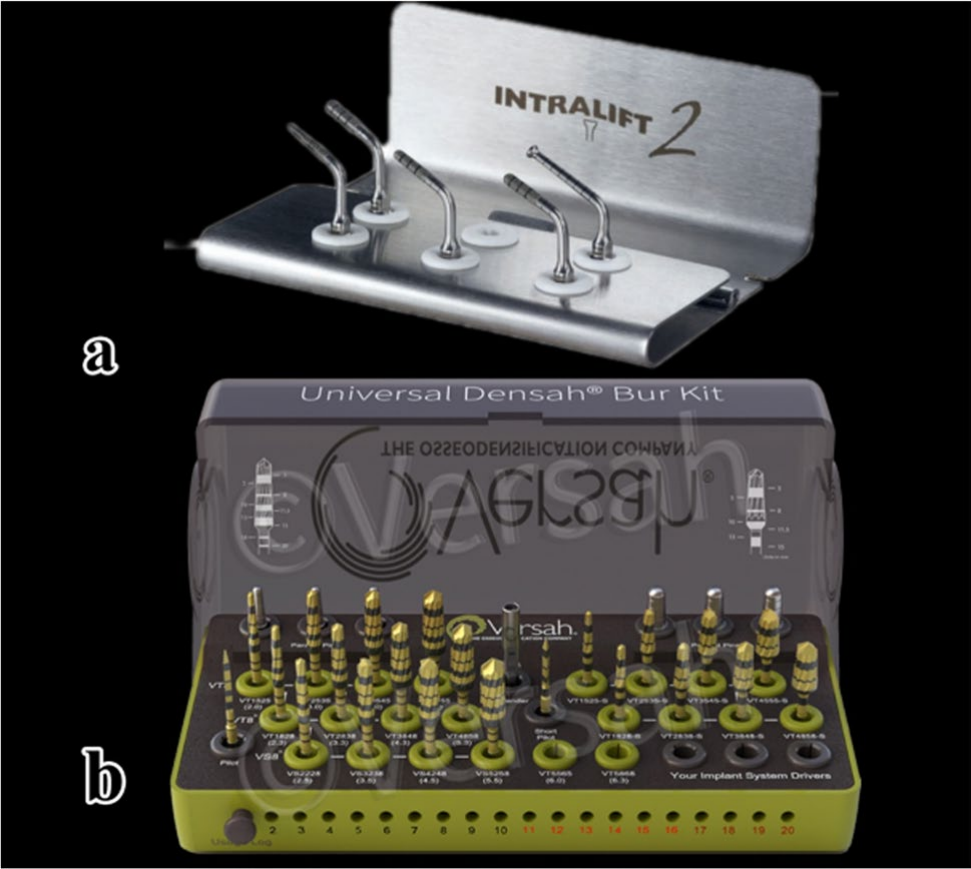
Displays: **a**) intralift kit tips ; **b**) Densah bur kit

*Piezoelectric device*: Intralift^®^ Kit (Acteon, France) [20] (Figure 1a).

*OD burs*: Densah^®^ osseodensification burs (Versah LLC, USA) [9] (Figure 1b).

*Stability measurement*: Osstell^®^ device (Integration Diagnostics Ltd., Sweden) for resonance frequency analysis (ISQ) [23].

*Bone graft*: Cancellous bone allograft (MinerOss Cancellous Particle size range: 300–1000 μm, BioHorizons, Birmingham, AL, USA) [21].

### Pre-surgical protocol

**Fig. 2:**
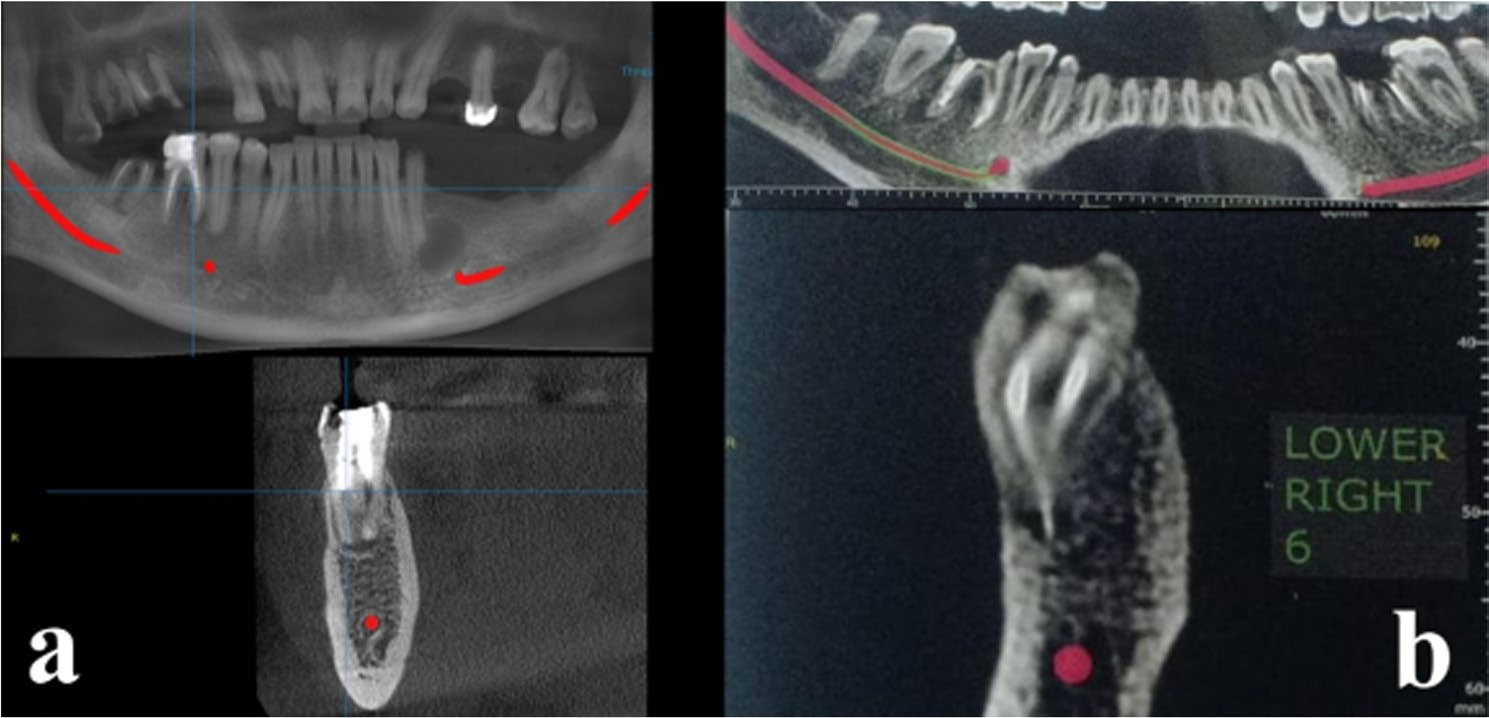
Display preoperative CBC xray **a**) group A PISP **b**) group B OD

Preoperative preparation included full mouth debridement using ultrasonic scalers and hand curettes, and oral hygiene instruction with 0.2% chlorhexidine mouthwash for seven days before surgery [24]. Preoperative CBCT scans Fig. 2A-B (94 kV, 12 mA) were acquired to assess bone quality, socket anatomy, and to plan implant dimensions [25] .

Preoperative bone density at each implant site was assessed using CBCT-derived gray values (Hounsfield Unit equivalents) measured at the planned implant location, in accordance with established methodology in implant site bone quality assessment [26]. CBCT scans were processed using OnDemand3D software (Cybermed Inc., Seoul, South Korea). A standardized rectangular region of interest (ROI) was placed at the projected implant body region, and the mean gray value within the ROI was recorded as the bone density value for each site.

Antibiotic prophylaxis consisted of 2 g amoxicillin–clavulanic acid (Augmentin^®^, GlaxoSmithKline, Brentford, UK) administered one hour pre-operatively [27]. Analgesics (50 mg diclofenac) were given preoperative.

### Surgical protocol

**Fig. 3:**
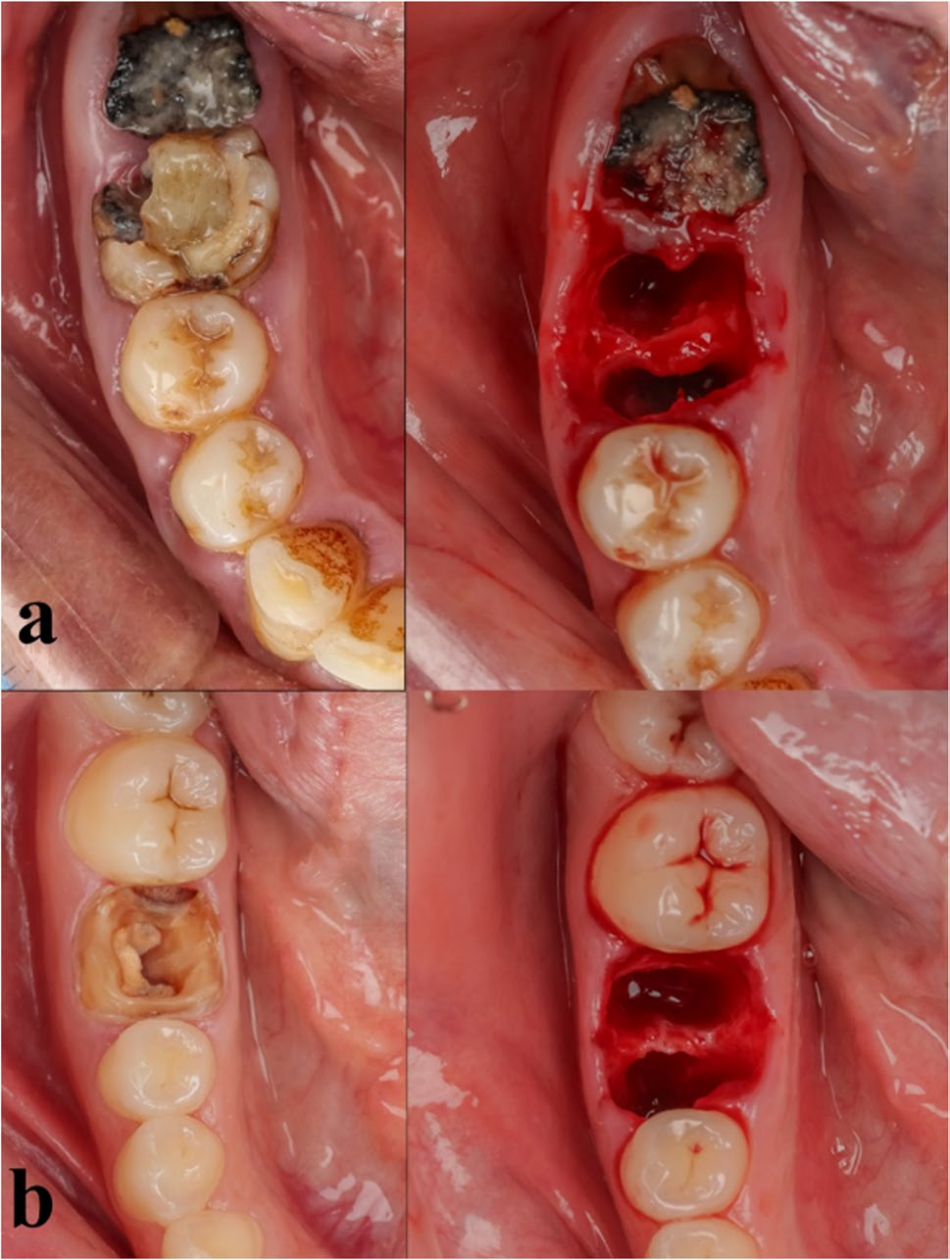
Displays atraumatic extraction and preoperative septal bone width **a**) PISP group **b**) OD group

Local anesthesia (4% articaine with 1:100 000 epinephrine) was administered. Teeth were extracted atraumatically using periotomes and sectioned to preserve the interradicular septum and buccal plate. (Fig. 3a-b).

Osteotomies for both were initiated with a pilot drill extending 1 mm beyond the planned implant length.

#### PISP group

**Fig. 4:**
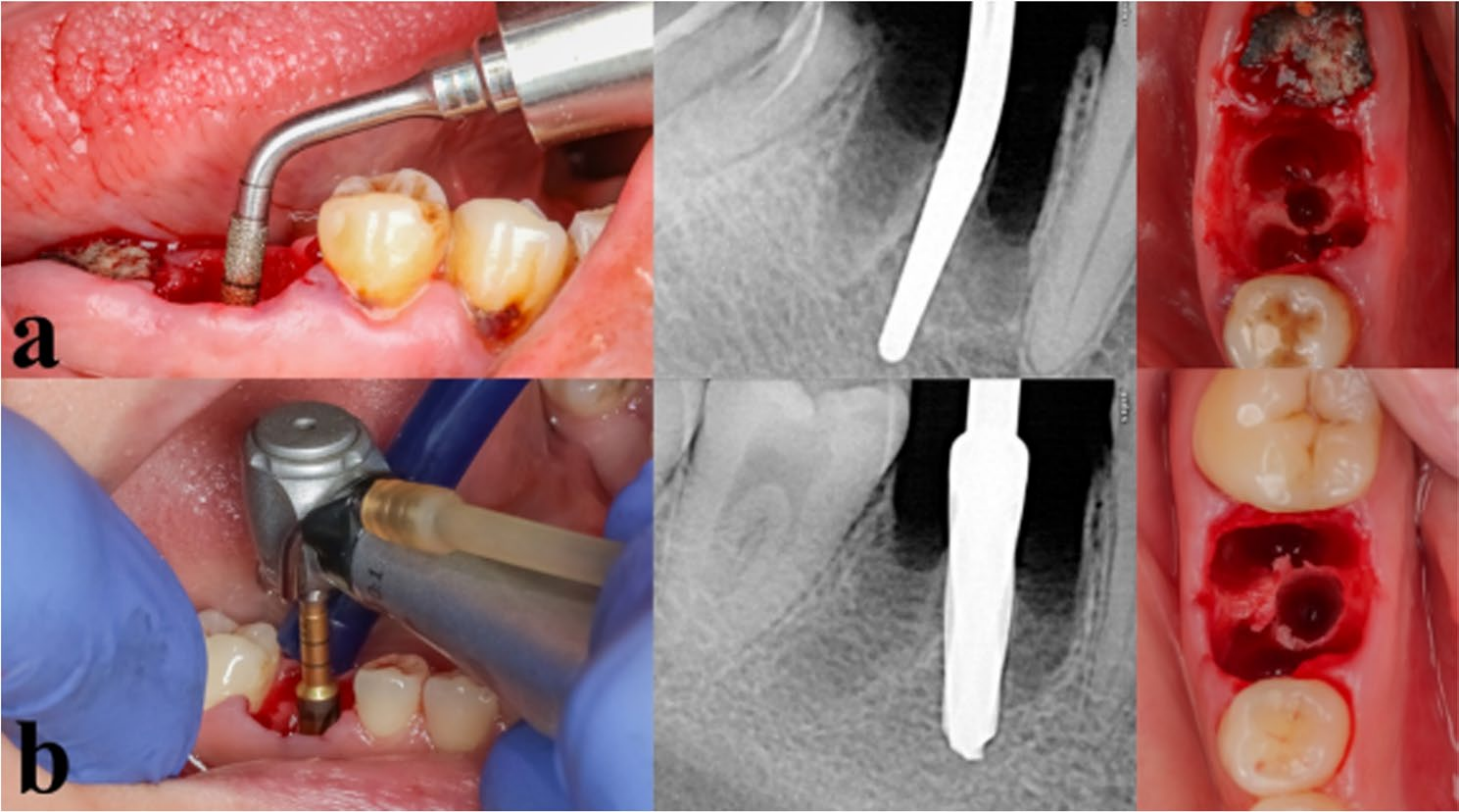
Displays the osteotomy preparation and achieved post operative septal bone expansion **a**) PISP group **b**) OD group

Osteotomy sites were prepared with the Intralift Kit’s diamond-coated tapered tips (TKW1 1.35 mm, TKW2 1.85 mm, TKW3 2.35 mm and TKW4 2.80 mm) under continuous irrigation (Fig. 4-a).

#### OD group

Osteotomy sites were prepared using Densah burs (VT1525 2.0 mm, VT1838 2.3 mm, VT2535 3.0 mm, VT2838 3.3 mm and VT3238 3.5 mm) in densifying mode counterclockwise motion and drilling speed (800–1500 rpm) under copious saline irrigation [12]. (Fig. 4-b)

### Osteotomy preparation time was recorded for each case starting from pilot drilling until completion of the final preparation

**Fig. 5:**
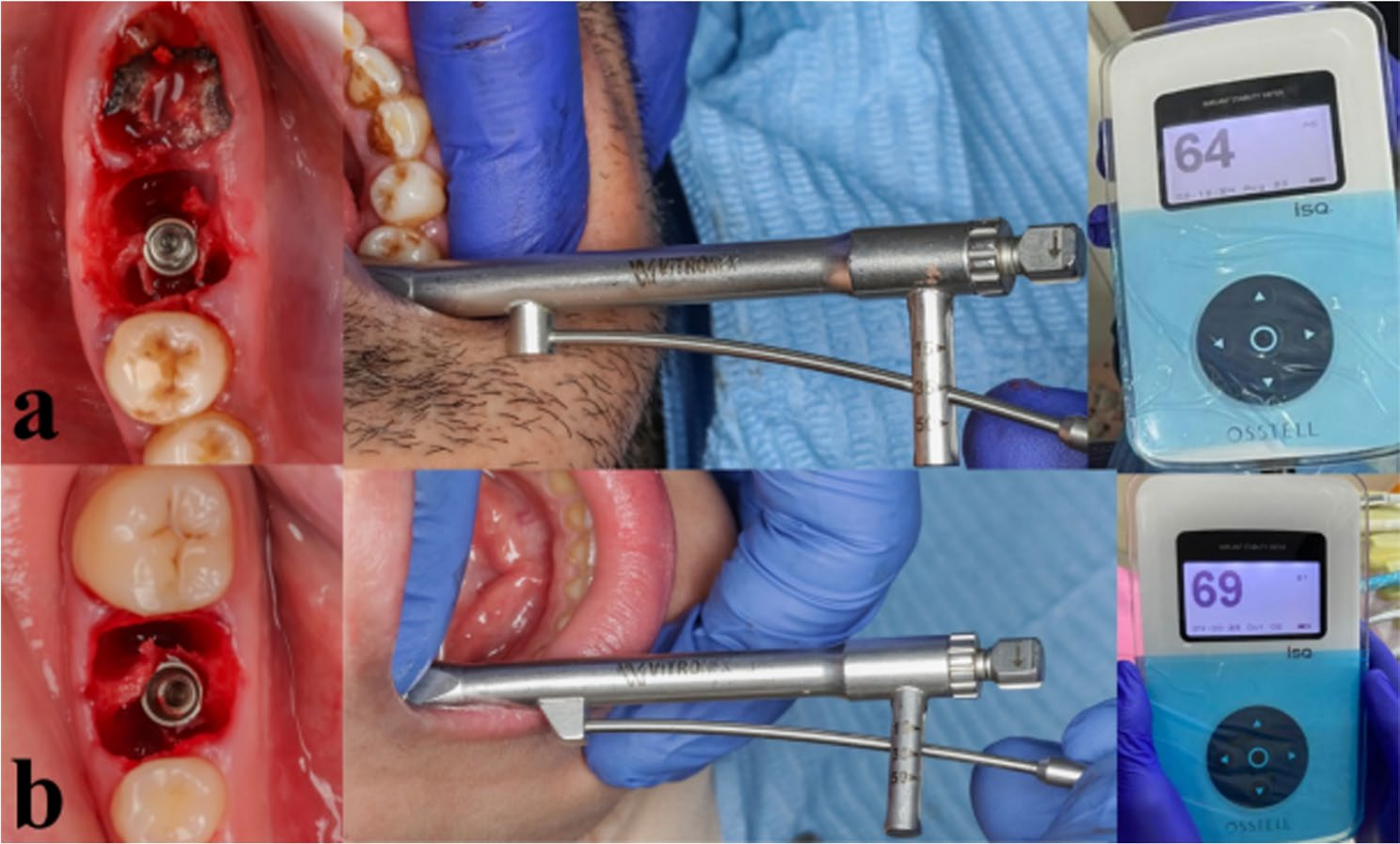
Displays implant placement, measuring insertion torque values by manual torque wrench, primary stability by Ostell

**Fig. 6:**
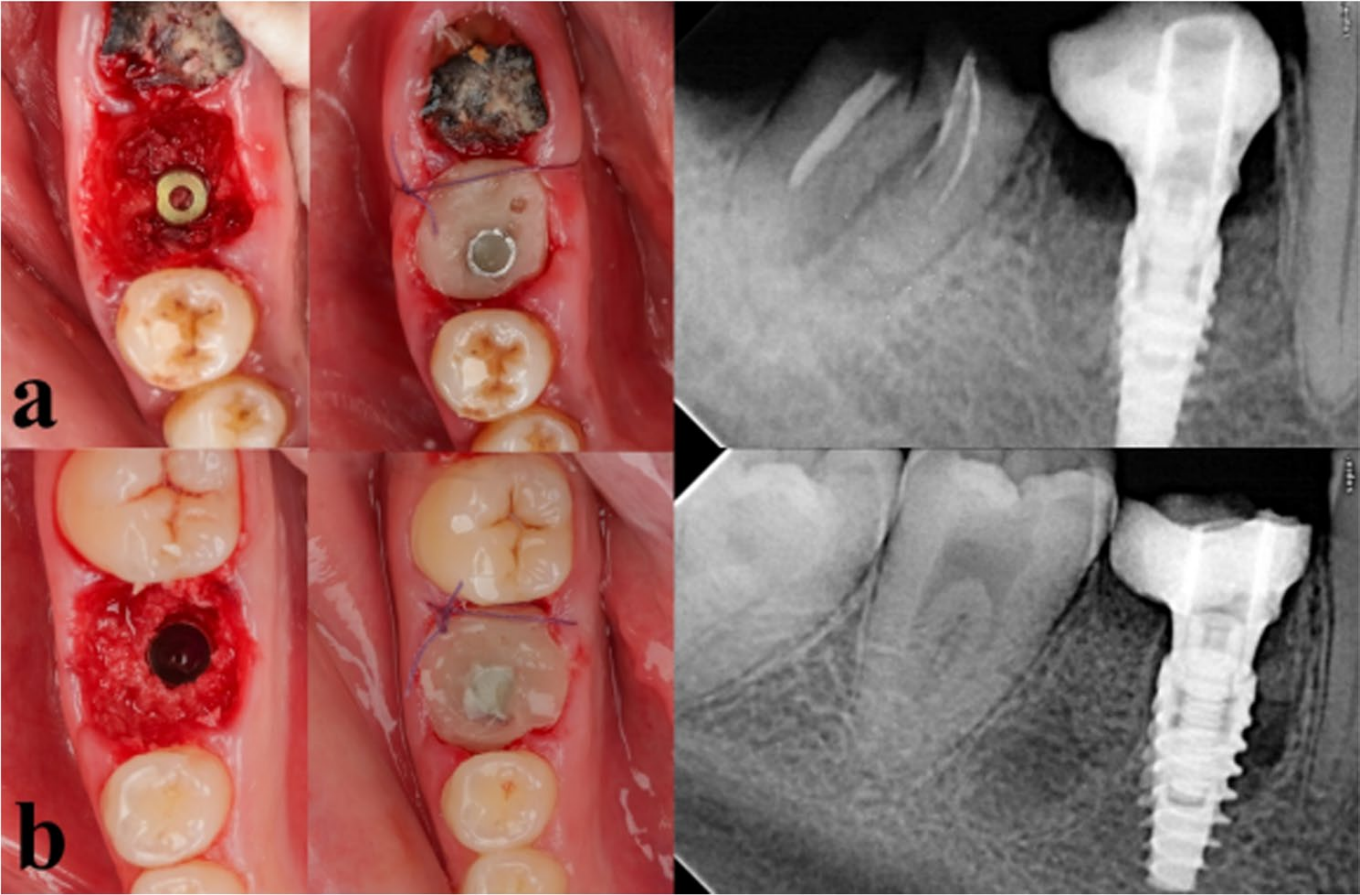
Displays jumping gap filling with allograft bone and sealing the sockets with custom made healing abutment and periapical xray immediate postoperative

Implants were placed subcrestal at level of septal bone using a manual torque ratchet, and peak insertion torque (Ncm) was documented (Figs. 5 and 6).

Primary stability (ISQ) was measured immediately after placement using an Osstell device. A standardized SmartPeg was used for all cases, and the probe was positioned 2–3 mm away from the magnet until a stable reading was obtained. Measurements were recorded in two directions (bucco-lingual and mesio-distal), and the mean value was used as the representative ISQ (Figs. 5 and 6).

Socket gaps were grafted with allograft bone, and customized healing abutments were placed to seal the site [28, 29]. (Figures 5 and 6).

### Postoperative care

Patients were instructed to avoid pressure on the surgical site, adhere to a soft diet for 14 days, and rinse with 0.2% chlorhexidine twice daily. Postoperative medications included diclofenac (cataflam 50) for analgesia and amoxicillin 500 mg TID for five days. All patients received a standardized medication protocol across both study groups. Postoperative pain was assessed using a visual analogue scale (VAS), where 0 indicated “no pain” and 10 indicated “sever pain ”. Patients reported their pain score once daily during the first postoperative week, and the values were recorded in the patient file. Follow-up after three months included ISQ measurement for secondary stability assessment.

**Fig. 7:**
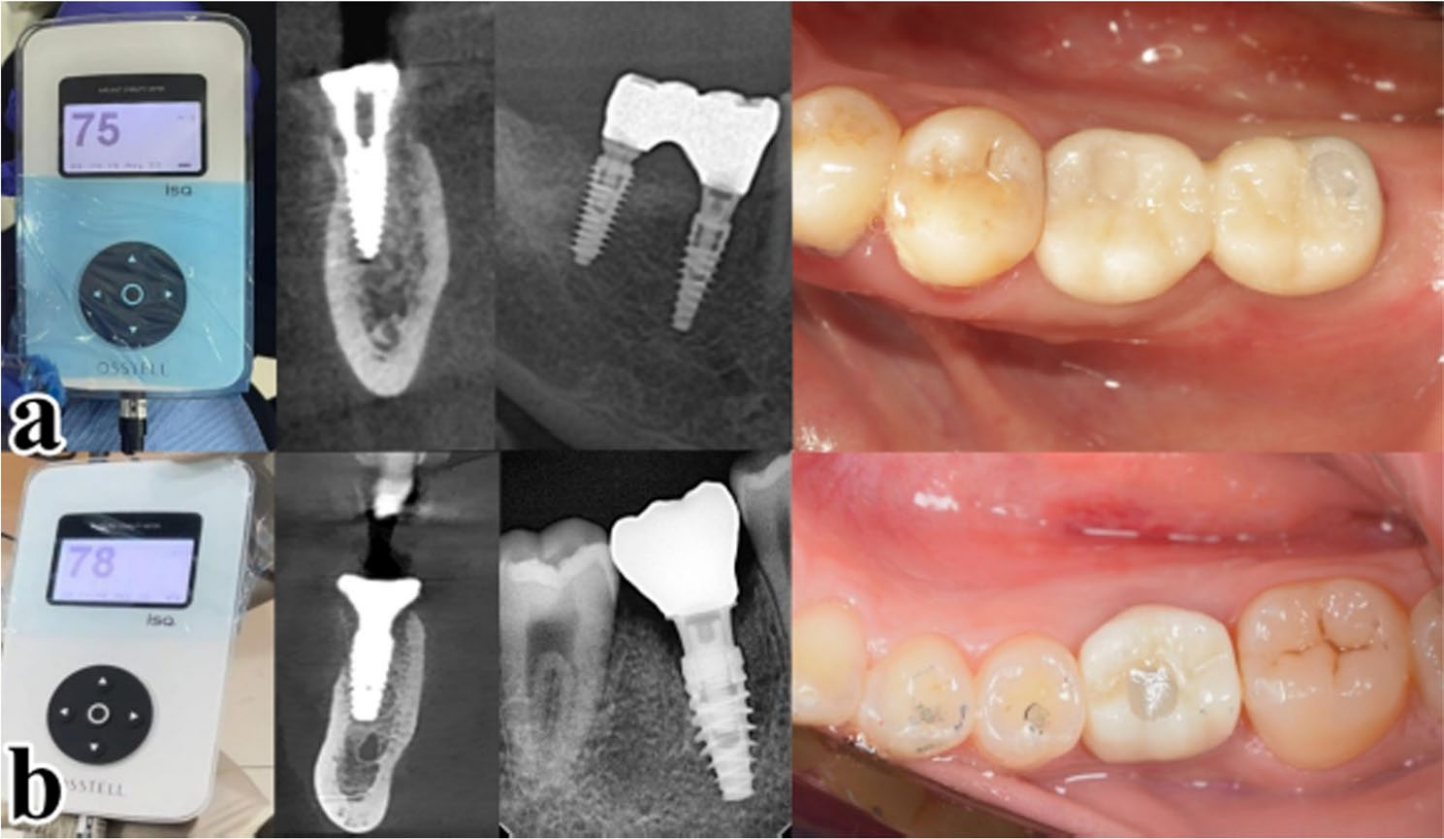
Display post operative secondary stability measure with Ostell, CBCT coronal cut, and periapical xray after 3 month and clinical pictures for screw retained crowns

Follow-up after three months included ISQ measurement for secondary stability assessment. (Fig. 7)

No intraoperative exclusions were required in this cohort. All enrolled patients successfully underwent the planned septal expansion and immediate placement.

### Sample size

Sample Size Calculation Based on Elgrany et al. [30], Sample size calculation was performed based on a previous study by Elgrany et al. [30], which reported mean primary stability values (ISQ) of 62.80 ± 2.25 for the PISP group and 68.30 ± 3.83 for the OD group. Using a two-sample t-test with an alpha level of 0.05 and 80% power to detect a mean difference of 5.5 in ISQ values (Effect size = 1.43), the required sample size was calculated as 9 patients per group. To account for potential dropouts, the sample size was increased to 10 patients per group (Total *N* = 20) [31]. 

### Statistical analysis

Data were analyzed using IBM SPSS Version 23 for Windows (Armonk, NY, USA). Normality was assessed using the Shapiro–Wilk test and Q–Q plots. Data were presented as mean and standard deviation, in addition to median, minimum, and maximum values where appropriate. Between-group comparisons for baseline bone density, primary ISQ, insertion torque, secondary ISQ, and osteotomy preparation time were performed using independent t-tests, while within-group comparisons were performed using paired t-tests. Percent change in stability was calculated using the formula: (Secondary stability – Primary stability) × 100.

Postoperative pain (VAS) was analyzed using non-parametric tests: between-group comparisons at each time point were performed using the Mann–Whitney U test, and within-group changes over time were evaluated using the Friedman test. A significance level of α = 0.05 was adopted for all analyses, and all tests were two-tailed.

**Figure:**
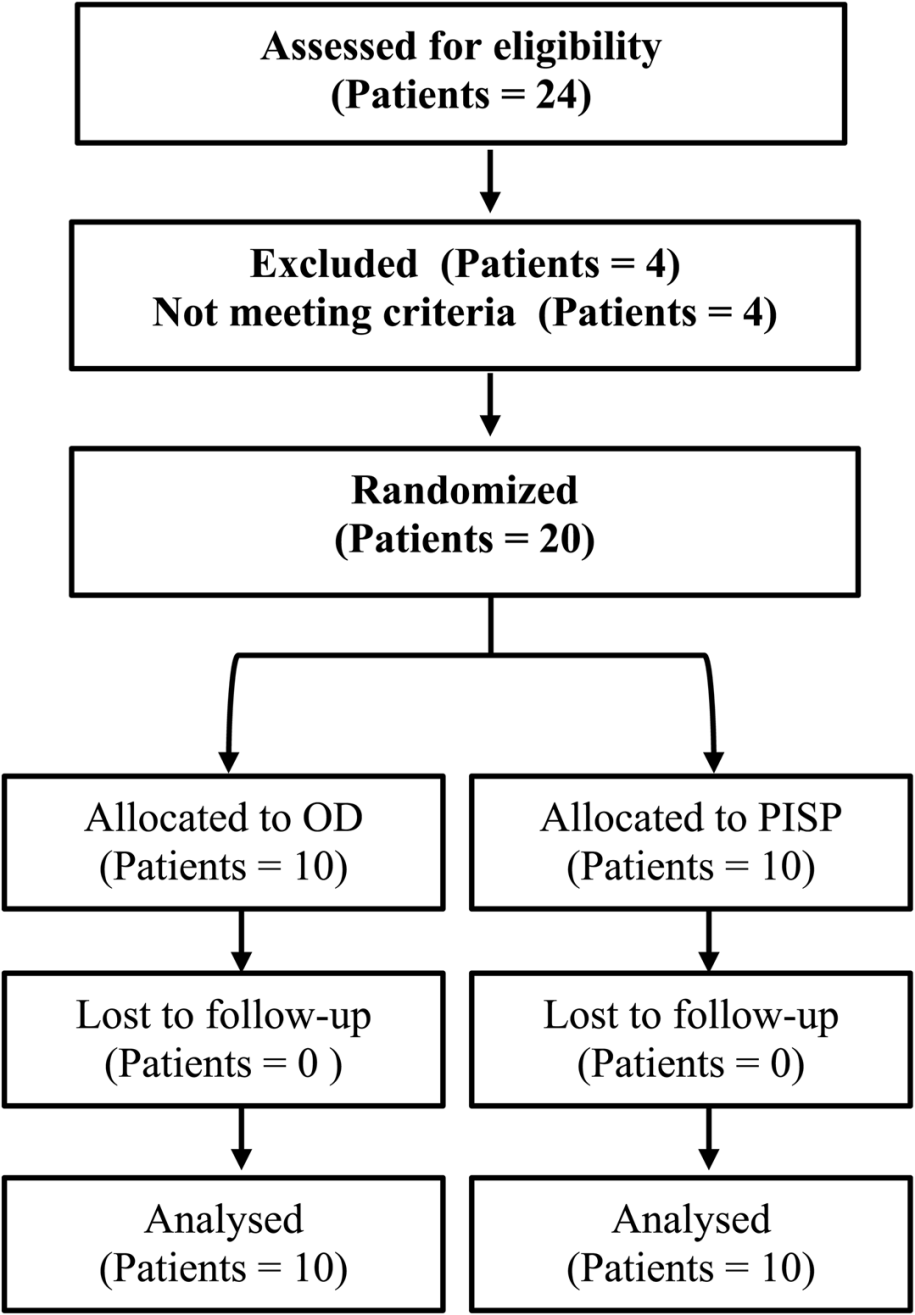
Consort diagram

## Results

All predefined feasibility criteria were met. Recruitment was completed within the planned timeframe, all 20 patients completed the full protocol with no dropouts, no serious adverse events were recorded, and both surgical techniques were successfully completed in all included sites. Both groups achieved clinically acceptable primary and secondary stability values. No postoperative complications were observed in either group, including early implant failure, infection, wound dehiscence, or neurosensory disturbance.

### Preoperative baseline bone density

Are presented in Table 1. Preoperative CBCT analysis revealed no statistically significant difference in baseline bone density between the PISP and OD groups (*p* = 0.614).

**Table 1:** Comparison of baseline bone density

	OD (*n* = 10)	PISP (*n* = 10)	*p* value
Baseline Mean ± SD	523.00 ± 35.92	515.00 ± 33.75	0.614
Median	525.00	515.00	
Min – Max	470.00–580.00	460.00–570.00	

Statistically significant difference at *p* value < 0.05

### The demographic characteristics of the participants

Are presented in Table 2. Twenty patients were included in the study and randomly assigned into two equal groups, with each patient receiving a single implant. The PISP group consisted of 5 females and 5 males with a mean age of 30.3 ± 1.5 years, while the OD group included 6 females and 4 males with a mean age of 29.4 ± 1.1 years, with no statistically significant difference between groups in terms of age or sex distribution (*p* > 0.05). In the PISP group, implants with a diameter of 3.7 mm were used, with lengths of 10 mm (40%), 11.5 mm (40%), and 13.5 mm (20%). In the OD group, implants with a diameter of 4.2 mm were placed, with lengths of 10 mm (30%), 11.5 mm (40%), and 13.5 mm (30%).All implants were successfully placed without intraoperative complications.

**Table 2:** Demographic characteristics and implant dimensions of the study population in both groups

Parameter	PISP (*n* = 10)	OD (*n* = 10)	*P*-value
Sex			0.682
Female	5 (50%)	6 (60%)	
Male	5 (50%)	4 (40%)	
Age (years)	30.3 ± 1.5	29.4 ± 1.1	0.138
Implant Dimensions			0.049*
3.7 × 10 mm	4 (40%)	–	
3.7 × 11.5 mm	4 (40%)	–	
3.7 × 13.5 mm	2 (20%)	–	
4.2 × 10 mm	–	3 (30%)	
4.2 × 11.5 mm	–	4 (40%)	
4.2 × 13.5 mm	–	3 (30%)	

No statistically significant differences were observed between groups for age or sex distribution

### Implant stability values

Are presented in Table 3. The OD group demonstrated slightly higher mean primary ISQ (69.25 ± 2.86) compared to the PISP group (66.75 ± 2.86), although the difference did not reach statistical significance (*p* = 0.082). Secondary ISQ values followed a similar pattern (78.50 ± 2.50 vs. 76.75 ± 2.05, *p* = 0.124). The percentage increase in stability was marginally greater in the PISP group (15.13 ± 4.81%) compared with the OD group (13.40 ± 1.17%), but this difference was also not significant (*p* = 0.310). Within group comparisons revealed significant improvements from primary to secondary stability for both techniques (*p* < 0.001).

**Table 3:** Comparison of implant stability between OD and PISP groups

		OD (*n* = 10)	PISP (*n* = 10)	*p* value
Primary	**Mean ± SD**	69.25 ± 2.86	66.75 ± 2.86	0.082
	**Median**	69.25	66.75	
	**Min – Max**	65.00–73.00	63.00–70.00	
Secondary	**Mean ± SD**	78.50 ± 2.50	76.75 ± 2.05	0.124
	**Median**	78.50	76.75	
	**Min – Max**	75.00–82.00	75.00–80.00	
*p* value		< 0.001*	< 0.001*	
% Increase	**Mean ± SD**	13.40 ± 1.17	15.13 ± 4.81	0.310
	**Median**	13.04	14.98	
	**Min – Max**	12.33–15.38	8.70–22.22	

Statistically significant difference at p value < 0.05

### Insertion torque values

Are presented in Table 4. The mean insertion torque was significantly higher in the PISP group (40.33 ± 3.24 Ncm; *p* = 0.004) compared to the OD group (35.00 ± 3.53 Ncm).

**Table 4:** Comparison of primary insertion torque between OD and PISP groups

		OD (*n* = 10)	PISP (*n* = 10)	*p* value
Primary Torque	**Mean ± SD**	35.00 ± 3.53	40.33 ± 3.24	0.004*
	**Median**	35.00	40.00	
	**Min – Max**	30.00–40.00	35.00–45.00	

Statistically significant difference at p value < 0.05

### The osteotomy preparation time

**Fig. 8:**
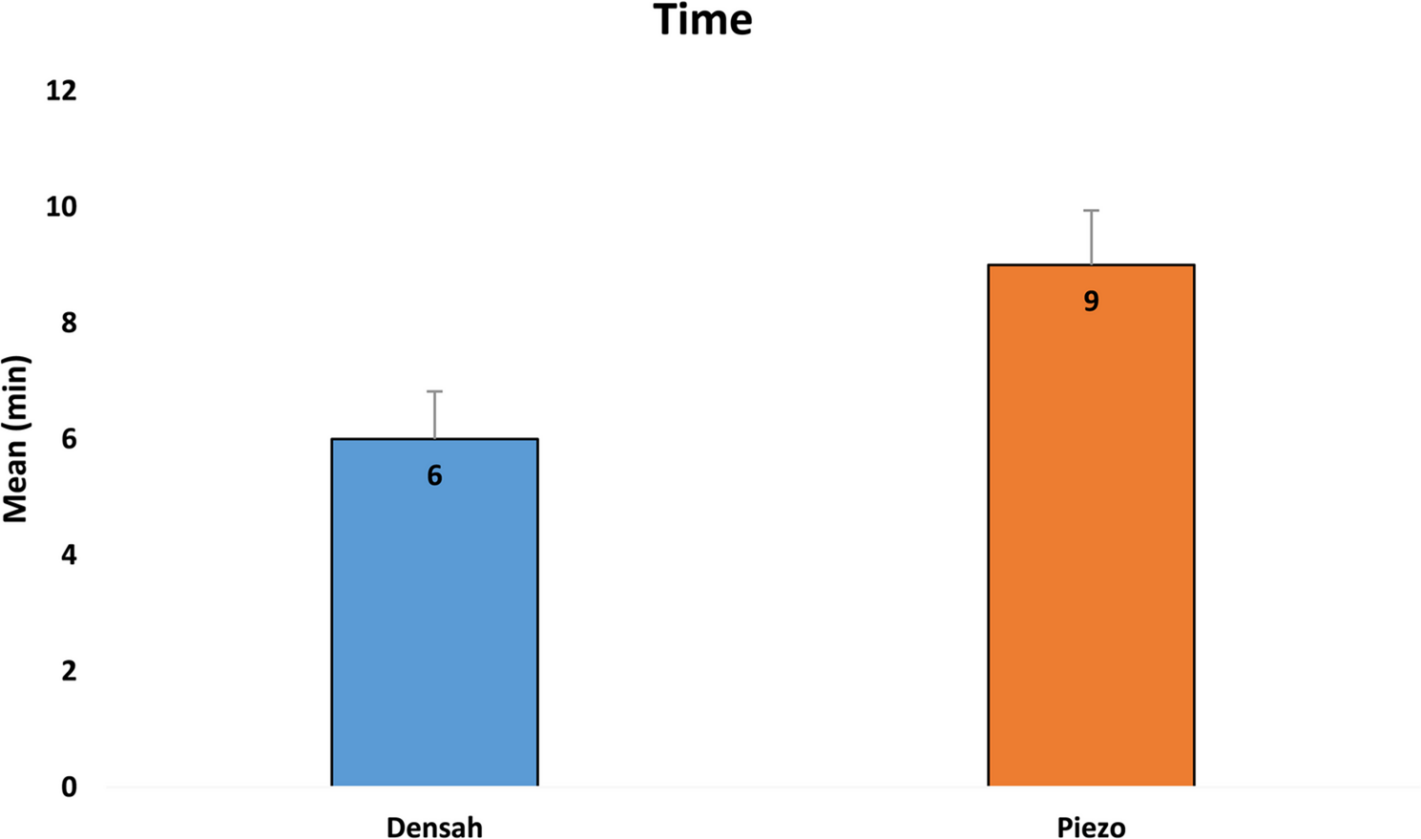
Comparison of osteotomy preparation time between PISP and OD groups

Are presented in Fig. 8. Osteotomy preparation time was significantly shorter in the OD group compared with the PISP group (*p* < 0.001). (Fig. 8)

### Postoperative pain (VAS)

**Fig. 9:**
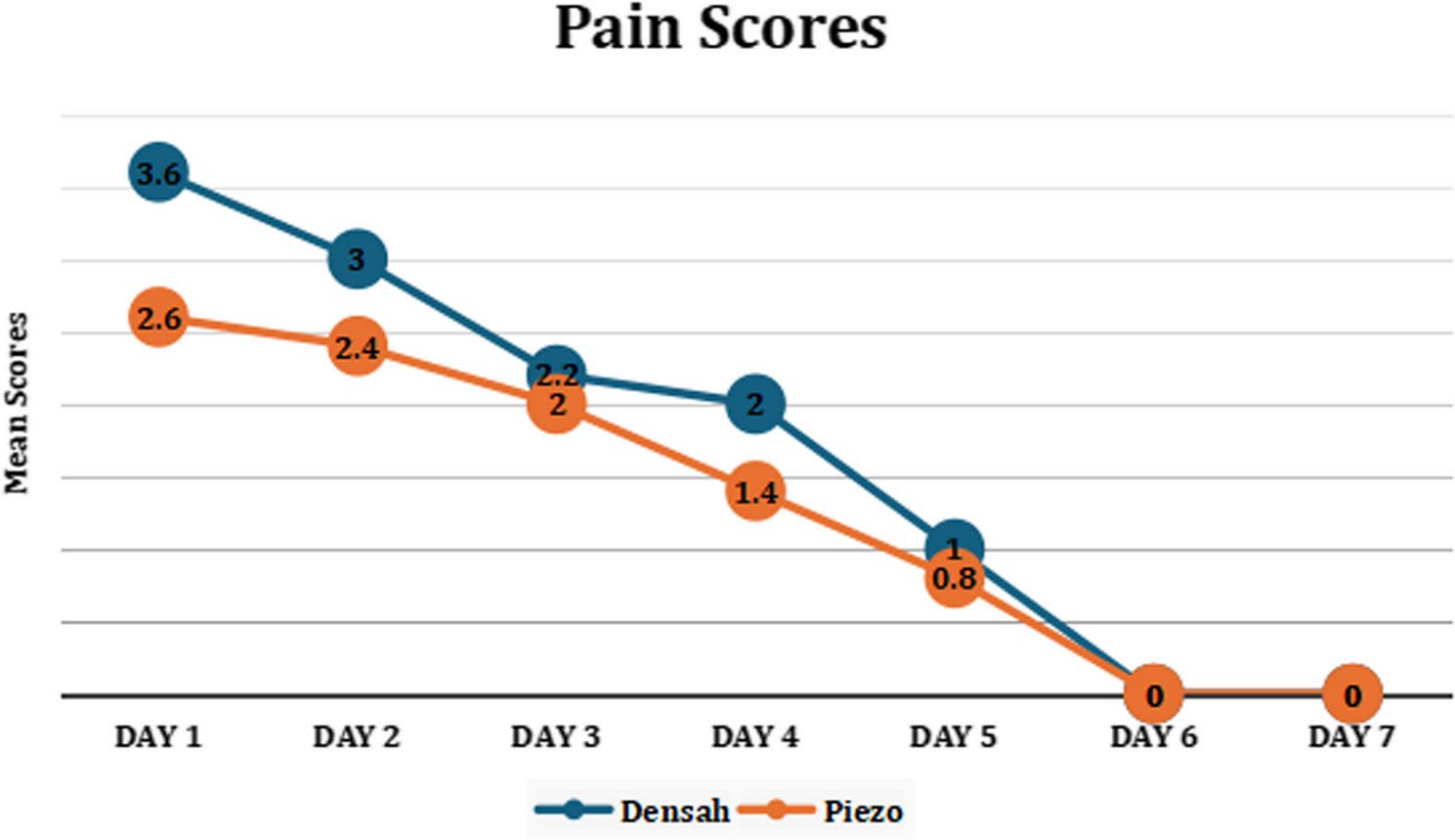
Postoperative pain scores (VAS) over the first postoperative week for both groups

Are presented in Fig. 9. VAS pain scores during the first postoperative week were significantly lower in the PISP group compared with the OD group (*p* < 0.05). Pain decreased progressively in both groups over time, with no complications reported.

## Discussion

Engaging the interradibular septal bone provides not only enhanced primary stability but also enables prosthetically driven implant positioning, allowing for placement within the long axis of occlusal forces [2]. This optimal biomechanical alignment reduces risks of prosthetic complications including screw loosening and implant fracture while simultaneously facilitating an ideal emergence profile that minimizes cantilever forces. These combined biomechanical and prosthetic advantages contribute significantly to the long term survival and success of implants [24, 32].

To best of our knowledge this study the first to investigate the application of the Intralift Kit for controlled septal expansion in mandibular molars. The protocol leveraged the kit’s unique design, utilizing an initial pilot drill to establish precise guidance for the subsequent sequential use of its angled, diamond-coated tips (1.35–2.80 mm) [20]. The system’s principal advantage in this anatomically complex region lies in its generation of high-frequency ultrasonic vibrations, which produce controlled hydrodynamic cavitation and micro vibrational stress. This mechanism facilitates a localized greenstick type plastic deformation through micro-fractures, resulting in modest yet clinically sufficient expansion while selectively emulsifying mineralized tissue and sparing adjacent neurovascular structures [16, 17, 33]. This selective cutting action minimizes the risk of iatrogenic injury compared to conventional rotary instruments.

In the present study, piezoelectric implant site preparation (PISP) using the Intralift Kit yielded a significantly higher mean insertion torque (40.33 ± 3.24 Ncm) compared to osseodensification (OD) with Densah^®^ burs (35.00 ± 3.53 Ncm; *p* = 0.004). This difference is most probably explained by the narrower final osteotomy created by the PISP protocol (2.8 mm), which results in higher mechanical interlocking during implant placement. These findings are in agreement with Samir et al. [20], who similarly reported higher insertion torque with piezoelectric site preparation and attributed it to the conservative osteotomy geometry. Importantly, both groups achieved mean insertion torque values well above the generally accepted clinical threshold (> 30–35 Ncm) considered sufficient for predictable primary stability and immediate implant placement. However, given the pilot feasibility design and limited sample size of the present study, the statistically significant differences observed in insertion torque must be interpreted cautiously as preliminary observations primarily intended to inform future trials.

Both PISP and OD techniques resulted in statistically comparable primary and secondary stability as measured by ISQ values, with no significant difference between the groups. The slightly higher, though non-significant, primary ISQ in the OD group can be attributed to the immediate mechanical advantage of its lateral bone compaction and spring-back effect, which create a denser peri-implant housing a mechanism well documented by Bergamo et al. [11]. The greater relative improvement in stability observed in the PISP group over the three-month healing period, while not statistically significant, is consistent with the biological advantages of piezoelectric surgery. As reported by Stacchi et al. and Gehrke et al. [34, 35], piezoelectric osteotomies can mitigate the early “stability dip” typically observed within the first weeks by promoting rapid new bone formation and reducing inflammatory response. This suggests that the immediate mechanical benefit of OD’s autocompaction is ultimately compensated by the enhanced biological environment created by PISP, resulting in secondary stability outcomes within clinically acceptable ranges in both groups at three months.

Implant stability was the main clinical focus of the present pilot RCT, assessed using resonance frequency analysis (RFA) expressed as the implant stability quotient (ISQ). RFA/ISQ is widely used as a non invasive clinical tool to monitor early healing and osseointegration and to support decisions related to loading protocols particularly during the early healing phase when stability changes are expected [34, 36, 37]. ISQ provides a standardized and repeatable assessment of stability over time, helping to monitor the transition from primary mechanical stability to secondary biologic stability [38–41].

Regarding osteotomy preparation time, the PISP required significantly longer preparation than OD. This difference can be attributed to the mechanism of piezoelectric surgery, which depends on micrometric, controlled cutting and sequential tip progression under continuous irrigation, a workflow that typically increases operative time compared with rotary-based site preparation. Similar findings have been reported in clinical studies comparing piezosurgery with conventional drilling, where piezoelectric preparation was associated with longer surgical duration while maintaining favorable stability outcomes [42, 43].

VAS pain scores during the first postoperative week were lower in the PISP group. This finding may be related to the less traumatic nature of ultrasonic site preparation with continuous irrigation. Similar reductions in early postoperative pain after piezoelectric/ultrasonic implant site preparation compared with conventional drilling have been reported in randomized clinical studies [42, 44].

Customized healing abutments provide critical prosthetic advantages in immediate implant protocols by actively guiding soft tissue healing and establishing optimal emergence profiles. Evidence confirms that anatomically shaped healing abutments better preserve peri-implant soft tissue contour and minimize buccal volume loss (Chokaree et al., 2024; Elgendi et al., 2024). In the present study, a silicone index was used to contour flowable composite resin over a temporary abutment, ensuring a smooth, biologically compatible interface with the surrounding soft tissues. This technique contributed to favorable mucosal health and emergence profile development during healing [45–47].

Measuring secondary stability at 3 months was intentionally selected to serve methodological goals to evaluate the early biological response to the surgical techniques and determine whether implant placement following septal expansion could achieve sufficient osseointegration to permit early loading [34, 48]. In addition, this follow up timing allowed us to isolate the effects of the surgical approach from prosthetic variables such as crown design, occlusal forces, and related complications that could confound implant stability outcomes [24, 32, 49, 50].

This pilot feasibility randomized clinical trial was conducted under standardized surgical and outcome measurement protocols, ensuring methodological consistency and strong internal validity. However, the modest sample size, single center design, strict age range, and absence of long term follow-up (≥ 12 months) to evaluate marginal bone loss and implant survival limit statistical power and generalizability. Therefore, the findings should be interpreted as preliminary and appropriate for a pilot feasibility study.

Given the difference in the final tip diameter of each system, which results in different final osteotomy diameters, varying implant sizes were necessarily utilized to prevent severe under preparation and the risk of excessively high insertion torque in the PISP group. While this approach was essential for surgical safety, we acknowledge that utilizing different absolute implant sizes may partially influence the comparative insertion torque and ISQ values.

Surgeon blinding was not feasible due to the inherent differences between the two osteotomy techniques; nevertheless, outcome assessment was performed under blinding to reduce measurement bias.

We recommend that future studies include larger multi-center samples and longer follow-up, including survival and marginal bone loss outcomes. Future trials should also incorporate validated pain and discomfort assessment tools, swelling/edema measurements, and soft-tissue healing indices, in addition to more comprehensive quantitative radiographic assessments. Further studies should evaluate a broader range of implant sizes and different implant systems to improve external validity. Additionally, future trials should standardize implant length and intraosseous implant depth across both groups to minimize any potential confounding influence on ISQ and BIC outcomes.

## Conclusions

The preliminary findings of this pilot feasibility study indicate that piezoelectric site preparation represents a clinically feasible alternative in anatomically complex posterior regions, achieving comparable primary and secondary stability outcomes to osseodensification. While this pilot trial provides favorable preliminary biomechanical data, future adequately powered multi-center studies with extended follow up after loading are needed to evaluate long term survival, marginal bone loss, and patient centered outcomes.

## Supplementary Information

Supplementary Material 1.

Supplementary Material 2.

## Data Availability

The datasets used and/or analyzed during the current study are available from the corresponding author, Dr. Hussein El. H. Hassan, upon reasonable request (Email: [dent.hussienelsaid91@gmail.com](mailto: dent.hussienelsaid91@gmail.com) ).

## Abbreviations

CBCT: Cone-beam computed tomography
IIP: Immediate implant placement
ISQ: Implant stability quotient
OD: Osseodensification
PISP: Piezoelectric implant site preparation
