# Supplementary material for: Implant stability following septal expansion in immediate mandibular molars: a pilot randomized clinical trial comparing piezoelectric surgery and osseodensification
Source: BMC Oral Health. 2026 Jul 17;26:1357. doi: 10.1186/s12903-026-09257-8 (PMC13422306; doi:10.1186/s12903-026-09257-8)
Supplement: Supplementary file 1 — Supplementary Material 1. [file 12903_2026_9257_MOESM1_ESM.docx]

**Hussien El. H. Hassan**

Faculty of Dentistry, Alexandria University, Alexandria, Egypt

Address Champollion St., Azarita, Alexandria, Egypt.

P.O. Box: 21523.

Email : [dent.hussienelsaid91@gmail.com](mailto:dent.hussienelsaid91@gmail.com)

Telephone number :+201148103883

ORCID iD: 0009-0002-0260-9269

Editor-in-Chief
BMC Medicine

Dear Dr Lee,

I am pleased to submit our manuscript entitled

**“Implant Stability Following Septal Expansion in Immediate Mandibular Molars: A Pilot Randomized Clinical Trial Comparing Piezoelectric Surgery and Osseodensification”**
for consideration for publication in *BMC Oral Health*.

This pilot randomized clinical trial investigated the **application of piezoelectric surgery with the Intralift® Kit** for immediate mandibular molar implant placement, compared with osseodensification using Densah® burs. The study aimed to provide preliminary data on implant stability outcomes associated with each technique and to evaluate the feasibility of septal expansion in this clinical context.

The research was conducted in accordance with the Declaration of Helsinki and approved by the Research Ethics Committee of Alexandria University (Approval No. 0716-06/2023; IORG008839). The trial was registered at ClinicalTrials.gov (NCT06957860).

A preprint version of this manuscript has been made available on **Research Square** to facilitate early dissemination and academic transparency. The version submitted here has been further revised and formatted according to *BMC Oral Health* submission guidelines.

To the best of our knowledge, this study contributes additional clinical evidence regarding the use of piezoelectric instruments for controlled septal expansion during immediate implant placement in mandibular molars. The findings are presented as preliminary data to guide future, larger-scale randomized clinical trials.

All authors have approved the final version of the manuscript and confirm that it has not been published or submitted elsewhere. There are no conflicts of interest or funding sources to declare.

We believe that the manuscript aligns well with the scope of *BMC Oral Health*, particularly within the field of implantology and clinical oral surgery, and may be of interest to readers seeking evidence on innovative implant site preparation techniques.

Thank you for your time and consideration.

**Sincerely,**
**Hussein El. H. Hassan**
